# Immunization status of children aged 12-23 months in Jonglei State, South Sudan: a cross-sectional epidemiologic study

**DOI:** 10.11604/pamj.2022.41.258.31637

**Published:** 2022-03-29

**Authors:** Jok Peter Mayom Jil

**Affiliations:** 1 World Health Organization, Juba, South Sudan

**Keywords:** Routine immunization, vaccine, low coverage, Jonglei, South Sudan

## Abstract

**Introduction:**

the immunization program focuses on reducing the morbidity and mortality associated with vaccine-preventable diseases. The purpose of the study was to determine the state of immunization coverage of children aged 12-23 months in Jonglei State, South Sudan, and to identify the factors that contribute to the low immunization coverage.

**Methods:**

a cross-sectional epidemiologic study was carried out between June and September 2020 using a predefined questionnaire based on the standard World Health Organization (WHO) Expanded Program on Immunization (EPI) protocol. A total of 385 women, 35 from 11 counties each of the Jonglei state who resided in the area for a minimum of 12 months, were randomly selected and individually interviewed. The immunization status of the child was verified either by health card or health card plus history recalls. Obtained data were subjected to statistical analysis.

**Results:**

only 17.7% and 27.5% of children were fully immunized as validated by health card and health card plus recall history, respectively. The most common reason for no immunization and partial immunization was a far distance of the health facility (24.9%) followed by lack of knowledge (23.1%). Based on the multivariate regression analysis of data verified by health card plus history recalls, age group of 25-29 years (OR=4.467 95% CI=1.112-1.795, p=0.000) and no knowledge of immunization (OR=1.578, 95% CI=1.438-4.579, p=0.000) significantly increased the odds of children being fully immunized, while Murle ethnic group(OR=0.083, 95% CI=0.008-0.849, p=0.036), delivery assistance by skilled birth attendance (OR=0.001, 95% CI=0.000-0.006, p=0.000) significantly decreased the odds of children being fully immunized.

**Conclusion:**

effective health education and easy access to health facilities and their utilization may significantly improve immunization in Jonglei, South Sudan.

## Introduction

The concept of immunization or vaccination is dated back to the late 1700s to early 1800s when an English doctor, Dr. Edward Jenner recommended using cowpox antigens as vaccination against smallpox, which was successful in culminating the smallpox disease from the world [1]. Today, immunization is considered one of the most effective public health intervention strategies adapted by the healthcare sector to reduce the mortality and morbidity associated with childhood infectious diseases. Several immunization programs have been developed by WHO and United Nations Children's Fund (UNICEF) in collaboration with the national immunization programs including Global Immunization Vision and Strategy (GIVS), global alliance for vaccines and immunization, Universal childhood immunization, GIVS and millennial development goals (MDGs) turned sustainable development goal (SDG), and the Global Vaccine Action Plan (GVAP) with an aim to decrease the incidence of vaccine preventable diseases (VPDs) and the morbidity and mortality rates associated with them [2]. Despite significant improvement in the overall child health and survival worldwide, most of the countries fail to reach the prescribed immunization targets at regional, state, and country levels [3], and several underdeveloped countries and developing countries still report high child mortality rates [4]. Therefore, there is an urgent need to improve the strategies of immunization plans and systems using evidence-based research. The new approaches must discuss measures to strengthen poor infrastructure, especially in developing countries, increase the manpower at grassroot levels and always make available the appropriate vaccine at the most affordable rates [5]. Immunization is one of the key priorities of the basic package of health and nutrition services in Independent South Sudan. According to the survey carried out in 2006, South Sudan reported a very low rate of immunization coverage of only 32% [6].

Post-independence, the first multi-year plan for expanded program on immunization (EPI) for 2007-2011 and 2011-2020 was implemented with a goal to achieve a population free from VPDs in the country [6]. It was recommended to vaccinate all infants by their first birthday as per the immunization regime suggested by the national and international standards to target the childhood diseases such as tuberculosis, poliomyelitis, diphtheria, pertussis, tetanus, measles, hepatitis B, and Hemophilus influenza infection, and to protect every newborn baby from neonatal tetanus. In South Sudan, despite the free vaccination programs offered by the government, the overall immunization coverage was substantially low in the subsequent EPI surveys conducted in 2011 [7] and 2017 [8] wherein, only 7.3% and 18.9% of children between the ages of12 and 23 months, respectively were fully immunized. Jonglei State is the largest state of South Sudan and one of those hard hits by the civil wars. According to the EPI survey conducted in 2011, fourteen counties including eight from the Jonglei State were reported to be having alarming low vaccination coverage with only 12% fully immunized children aged 12-23 months with a low rate (<20%) of individual vaccinations [6]. Moreover, the routine immunization dataset available in the District Health Information Software (DHIS-2) for the period of 2015 to 2020 indicates critical low coverage between 10% and 20% with unclear reasons for these extremely below coverage rates than the minimal target limit of 80% at counties level. Due to the lack of specific research, little is known about the factors responsible for continuous low routine immunization coverage in Jonglei State. Therefore, the present study was conducted with objectives to determine the state of immunization coverage of children aged 12-23 months in Jonglei State, South Sudan, and to identify maternal, child, and health-related factors that contribute to the low immunization coverage. The results of the survey will guide to formulation of recommendations and evidence-based interventions to increase the demand for immunization, which in turn can be used to improve the immunization coverage at county and state levels to achieve the national target.

## Methods

**Study design and population:** this cross-sectional epidemiologic study was conducted between June and September 2020 to ascertain the immunization status of children between the ages of 12 and 23 months in Jonglei State, the largest state located in the Eastern region of South Sudan. Mothers or caregivers of children between 12 and 23 months of age who gave birth or resided in the area for a minimum of 12 months duration were included in the study. Those mothers who were unable to give accurate reasons for low routine immunization were excluded. Since the degree of variability was unknown due to large population size, the sample size was determined by using the statistical formula of Fisher,


$$N=\left(\frac{Z^{2}pq}{d^{2}}\right)$$

where, z = 1.96 (95% level of confidence); d = 0.05 (level of precision at 5% level of significance); p = 0.5 (estimated immunization coverage) and q = 0.5(1-p). A sample size of 384.16 adjusted to 385 was derived. For the purpose of the study, 35 women or caretakers of children aged 12-23 months were randomly and equally selected from the lowest level of administration namely Payams and Bomas of 11 counties, each by allotting a specific number to each household and picking a random number. Andersen´s behavioral model, a questionnaire comprised of six parts having either yes or no responses or multiple choices, was formulated. The initial part consisted of details of the state, county, Payam, Boma, name of health facility, interviewer´s name, and code. Part one comprised of questions related to the sociodemographic characteristics of the mother including, age, ethnicity, religion, residence status, level of education, occupation, and distance from the health facility.

The second part covered the maternal health status before, during, and after the delivery. The third part of the questionnaire focused on the mother´s/caretaker´s knowledge on child immunization. Demographic characteristics of a child were recorded in the fourth part. The final part of the survey covered the reasons for partial or non-immunization. A pilot study was conducted at Pariak Primary Health Care Centre (PHCC) (located in the rural area) and Bor State Hospital (located in the urban setting) in Bor South County to understand the numerical arrangement of the questionnaire, time to complete the research, ease of response by participants, validity, and reliability. Based on this, unclear questions, wordings, sequences, and those questions that the participants considered not easy to talk about were rephrased or removed. Local interviewers were selected for remote locations and were provided with structured interview questions and a signature on the volunteer agreement form was obtained. All the selected mothers or caregivers of children aged 12-23 months signed informed consent prior to the interview. The individual data obtained from the interview and responses to the questionnaire were treated as confidential. The recorded data were thoroughly checked for errors and corrected accordingly for completeness. The details of immunization were verified with the health card of the child. The details were verified by the mother or caretaker´s recall of immunization status and by checking for the Bacillus Calmette-Guérin (BCG) scar on the left arm. A child who had been administered with all recommended vaccines based on EPI of South Sudan viz, a single dose of BCG vaccine, four doses of Oral Polio Vaccine (OPV), three doses of pentavalent vaccines, a single dose of inactivated Poliovirus Vaccine (IPV), and measles vaccine each was considered fully immunized. While those children who had started immunization sessions at a health facility but did not complete the prescribed doses, especially the Penta 3 and Measles doses at recommended ages, were considered partially immunized. Those children with no record of any vaccine administration were considered not immunized or not vaccinated.

**Statistical analysis:** all the recorded data were entered in Microsoft Excel and then imported to the Centre for Disease and Control (CDC) statistical software epidemiological information (EPI-Info) and Statistical Package for Social Sciences (SPSS) version 20.0 for statistical analysis. Univariate analysis for all the available variables in the questionnaire were carried out using descriptive statistics and results were reported as frequency and percentages. Pearson’s chi-square test was used to study the relationship between the immunization coverage (dependent variable) and sociodemographic factors of mother and child, maternal health factors, knowledge on immunization, and reasons for partial and non-immunization (independent factors). Regression analysis was used to estimate the strength of association between the predictors of full immunization and immunization coverage. The significance level was set at a 95% confidence interval, and statistical significance was defined at a p-value of 0.05.

**Ethical approval:** Department of Research and Ethical committee of Universita Telematica Internationale (Uninettuno University), Rome, Italy approved the study. Written permission was obtained from the State Ministry of Health and Environment, Jonglei to carry out research in the state. Informed Consent from mothers or caretakers of the children targeted was sought before enrolling participants to the study.

## Results

**Figure 1:**
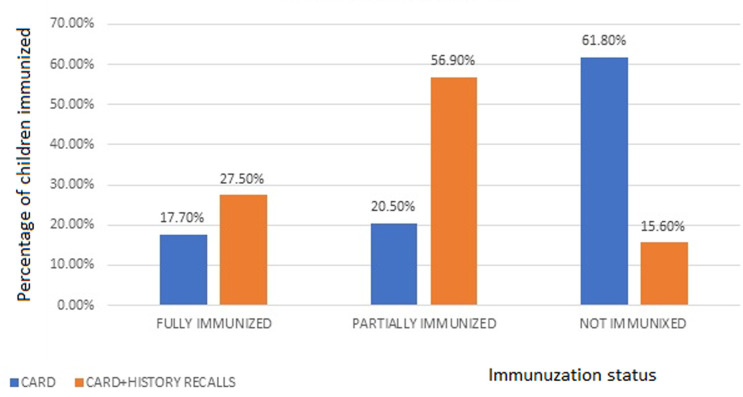
status of immunization in children aged 17-23 months

**Figure 2:**
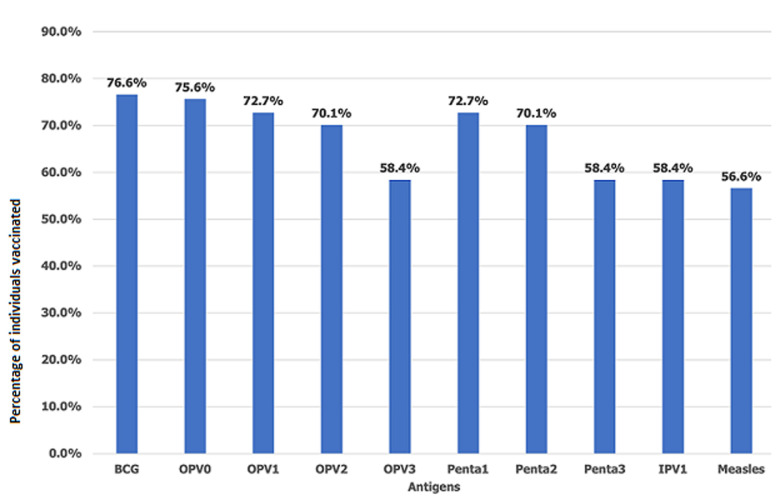
rate of individual vaccinations in the study population

In the present study, the status of immunization using cards and cards plus history recalls are as follows: fully immunized (17.7% vs 27.5%), partially immunized (20.5% vs 56.9%), and not immunized (61.8% vs 15.6%) (Figure 1). Similarly, specific vaccine coverage reported in the study is depicted in Figure 2. The frequency of vaccination administered at initial visits (>70%) was higher than those administered at a later date (50-60%). The mean age of the participants was 27.49 years (range 20-44 years). Most of the participants (39.2%) belonged to the age group of 25-29 years, 49.1% belonged to the ethnic group of Nuer, and almost all (99.5%) participants were Christians. While 97.9% of families lived in a village, 2.1% lived in the Internally Displaced Camps or Protection of Civilians site (IDPs/PoC). Around 93.2% of participants had not received any form of formal education, and 95.6% were housewives. Around 60.5% of them had to walk more than 60 minutes to reach a health facility (HF). The majority (60.3%) of the mothers/caretakers did not Attend Antenatal Care (ANC) services during pregnancy and 60.5% had not taken tetanus vaccination during pregnancy. Around 60.5% of the children were delivered at home, 61.8% of mothers were assisted by Traditional Birth Attendants (TBAs) and 66% of them had Postnatal Care (PNC) follow-up. The majority (64.9%) of them had not heard about immunization, while only 135 (35.1%) participants had some knowledge about child immunization services. The source of information was the husband (66.7%) and community mobilizers (33.3%). Around 60.3% of children were aged 12-17 months, and 39.7% were aged 18-23 months. The study population consisted of 51.9% males and 48.1% females. Around 39.2% of children were found to be ill two weeks before the vaccination schedule (Table 1). Bivariate analysis verified by card only and card plus history recalls showed higher immunization status in children of young mothers (<30 years) vs older mothers, Jie ethnic group vs other ethnic groups, children living IDP/PoC than those living in the village, educated mothers vs mothers with no formal education, unemployed mothers vs working mothers, and short travel time to (HF) as compared to longer travel time.

**Table 1:** frequency distribution of Socio-demographic characteristics of mother and child, maternal health factors and immunization knowledge

Variables	Dependent variable	Frequency	Percentage
**Socio-demographic characteristics of mother/caretaker**			
**Age**	20-24	123	31.9
	25-29	151	39.2
	30-34	71	18.4
	35-39	30	7.8
	>40	10	2.6
**Ethnicity**	Anyuak	35	9.1
	Dinka	126	32.7
	Jie	2	0.5
	Kachipo	3	0.8
	Murle	30	7.8
	Nuer	189	49.1
**Religion**	Christian	383	99.5
	Muslim	2	0.5
**Residence status**	IDP/PoC	8	2.1
	Own village	377	97.9
**Education**	Primary	19	4.9
	Secondary	6	1.6
	College/University	1	0.3
	No education	359	93.2
**Employment**	No employment	368	95.6
	Formal employment	17	4.4
**Distance to Health facility**	30 minutes	54	14
	60 minutes	98	25.5
	> 60 minutes	233	60.5
**Maternal health related factors: ANC attendance**	Yes	153	39.7
	No	232	60.3
**Number of visits**	One	73	47.7
	Two	68	44.4
	Three	12	7.8
	Four	0	0
**TT vaccination**	Yes	152	39.5
	No	233	60.5
**No. of doses**	One dose	40	26.3
	>One dose	112	73.7
**Delivery place of child**	Home	233	60.5
	Health facility	152	39.5
**Midwife**	Traditional birth attendance	238	61.8
	Skilled birth attendance	147	38.2
**Postnatal services**	Yes	131	34
	No	254	66
**Demographic characteristics of child**			
**Age**	12-17 months	232	60.3
	18-23 months	153	39.7
**Sex**	Male	200	51.9
	Female	185	48.1
**Child sick 2 weeks before vaccination**	Yes	151	39.2
	No	234	60.8
**Maternal knowledge on child immunization services**	Yes	135	35.1
	No	250	64.9
**Source of information**	Husband	90	66.7
	Community mobilizer	45	33.3
	Others (relatives, neighbors etc)	0	0
**Immunization session**	One	30	22.2
	Two	43	31.9
	Three	51	37.9
	Four	10	7.4
	Five	1	0.7
	Don't know	0	0
**Immunization starting age**	At birth	134	99.3
	After one month or more	1	0.7
	Child not immunized	0	0
	Don't know	0	0
**Age to complete vaccination**	Before one year (9 months)	131	97
	1 Year or more	4	3
	Don't know	0	0

These associations were statistically significant (p<0.005). The rate of full immunization was higher in the younger children than the older children (p=0.000). None of the girl children was fully immunized in this study, while 34.0% of male children were fully immunized (p=0.000). Children who were ill before the 2 weeks of immunization schedule were more likely to get vaccinated as compared to those who were never ill (p=0.000). Mothers who had availed ANC, PNC services, and those who had taken tetanus toxoid (TT) vaccine were more likely to vaccinate their children as compared to those who have not visited antenatal services(p=0.000). Immunization coverage was higher in children born at home and assisted by TBA (p=0.000). The rate of immunization was higher in children with mothers having knowledge of immunization programs (p=0.000). Knowledge gained from the husband had a higher impact on the rate of immunization as compared to community mobilizers (p=0.000) (Table 2). Univariate logistic regression analysis based on the card only and card plus history recalls showed that age of the mother (p<0.05), education (p=0.015), distance from a health facility (p=0.000), antenatal visits (p=0.001), and postnatal visits by community worker (p=0.000)and gender of the child (p=0.036) decreased the odds of immunization (Table 3). Multivariate regression analysis of immunization status with card only showed that mothers aged 25-29 (p=0.000), no knowledge on immunization (p=0.000), and travel time of 60 minutes (p=0.001) decreased the odds of full immunization. In case of partial immunization, mother's age group of 25-29 years (p=0.000) and 30-34 years (p=0.000), travel time of >60 minutes (p=0.000), no ANC visits (p=0.000), delivery at a health facility (p=0.000), delivery assistance by skilled birth attendant (SBA) (p=0.000), no postnatal visit by community health worker (p=0.015), no knowledge on immunization (p=0.000), child aged 18-23 months (p=0.000) and female child (p=0.000) decreased the odds of immunization.

**Table 2:** maternal socio-demographic factors associated with immunization coverage verified by card only and card and history recalls

Variables	Dependent Variable	Card only				Card plus history			
		NI	PI	FI	p-value	NI	PI	FI	p-value
**Socio-demographic characteristics of mother/caretaker**									
**AGE**	20-24	20	42	61	0	0	34	89	0
	25-29	110	34	7	0	3	131	17	0
	30-34	68	3	0	0	19	52	0	0
	35-39	30	0	0	0	28	2	0	0
	>40	10	0	0	0	10	0	0	0
**Ethnicity**	Anyuak	24	6	5	0	6	20	9	0.005
	Dinka	33	33	12	0	19	76	31	0.005
	Jie	0	0	2	0	0	0	2	0.005
	Kachipo	0	1	2	0	0	0	3	0.005
	Murle	25	5	0	0	8	21	1	0.005
	Nuer	108	34	47	0	27	102	60	0.005
**Religion**	Christian	237	79	67	0.439	60	218	105	0.705
	Muslim	1	0	1	0.439	0	1	1	0.705
**Residence status**	IDP/PoC	0	0	8	0	0	0	8	0
	Own village	238	79	60	0	60	219	98	0
**Education**	Primary	0	0	19	0	0	0	19	0
	Secondary	0	1	5	0	0	0	6	0
	College/University	0	0	1	0	0	0	1	0
	No Education	238	78	43	0	60	219	80	0
**Employment**	No Employment	221	79	68	0.004	48	214	106	0
	Formal Employment	17	0	0	0.004	12	5	0	0
**Distance to Health facility**	30 Minutes	1	4	49	0	1	0	53	0
	60 minutes	13	66	19	0	0	45	53	0
	> 60 minutes	224	9	0	0	59	174	0	0
**Maternal Health related factors**									
**ANC attendance**	Yes	16	69	68	0	0	47	106	0
	No	222	10	0		60	172	0	0
**Number of visits**	One	0	13	60	0	0	2	71	0
	Two	10	50	8	0	0	37	31	0
	Three	6	6	0	0	0	8	4	0
**TT Vaccination**	Yes	16	68	68	0	0	46	106	0
	No	222	11	0	0	60	173	0	0
**No. of doses**	One Dose	0	1	39	0	0	0	40	0
	>One Dose	16	67	29	0	0	46	66	0
**Place of Delivery**	Home	94	71	68	0	3	124	106	0
	Health Facility	144	8	0	0	57	95	0	0
**Midwife**	Traditional Birth Attendance	101	69	68	0	3	131	104	0
	Skilled Birth Attendance	137	10	0	0	57	88	2	0
**Postnatal services**	Yes	8	55	68	0	0	25	106	0
	No	230	24	0	0	60	194	0	0
**Demographic characteristics of child**									
**Age**	12-17 months	87	77	68	0	0	126	106	0
	18-23 months	151	2	0	0	60	93	0	0
**Sex**	Male	58	74	68	0	0	94	106	0
	Female	180	5	0	0	60	125	0	0
**Child sick 2 weeks before vaccination**	Yes	23	60	68	0	0	53	98	0
	No	215	19	0	0	60	166	8	0
**Maternal knowledge on child immunization services**									
**Knowledge of immunization services**	Yes	12	57	66	0	0	33	102	0
	No	226	22	2	0	60	186	4	0
**Source of information**	Husband	0	26	64	0	0	5	85	0
	Community mobilizer	12	31	2	0	0	28	17	0
**Immunization session**	One	0	0	30	0	0	0	30	0
	Two	0	11	32	0	0	1	42	0
	Three	7	40	4	0	0	26	25	0
	Four	4	6	0	0	0	5	5	0
	Five	1	0	0	0	0	1	0	0
**Immunization starting age**	At birth	12	56	66	0.502	0	32	102	0.502
	After one month or more	0	1	0	0	0	1	0	0
**Age to complete vaccination**	Before one year (9 months)	12	53	66	0.06	0	31	100	0.06
	1 Year or more	0	4	0	0	0	2	2	0

**Table 3:** predictors of immunization coverage

	Card only		Card and history recall	
Variable	OR (95% CI)	P value	OR (95% CI)	P value
Intercept	-0.645 (-1.144, -0.146)	0.011	-0.213 (-0.699, 0.273)	0.389
Age of mother	0.060 (0.002, 0.119)	0.044	0.344 (0.287, 0.401)	0
Ethnicity	0.000 (-0.016, 0.017)	0.963	0.007 (-0.010, 0.023)	0.421
Residence	0.216 (-0.041, 0.473)	0.1	0.127 (-0.123, 0.378)	0.318
Education	0.075 (0.015, 0.135)	0.015	0.023 (-0.036, 0.081)	0.448
Occupation	0.043 (-0.132, 0.219)	0.627	0.034 (-0.137, 0.205)	0.693
Distance to HF	0.561 (0.458, 0.663)	0	0.068 (-0.032, 0.168)	0.184
ANC attendance	0.312 (0.137, 0.487)	0.001	-0.006 (-0.177, 0.164)	0.942
Delivery place	-0.108 (-0.316, 0.100)	0.306	-0.043 (-0.245, 0.160)	0.678
Delivery assistance	-0.065 (-0.252, 0.123)	0.499	-0.051 (-0.234, 0.132)	0.583
Postnatal service	0.317 (0.147, 0.488)	0	0.617 (0.450, 0.783)	0
Knowledge	-0.018 (-0.176, 0.139)	0.819	0.011 (-0.143, 0.164)	0.891
Age of child	0.111 (-0.037, 0.259)	0.14	0.098 (-0.046, 0.242)	0.182
Gender of child	-0.064 (-0.216, 0.088)	0.407	-0.158 (-0.306, -0.010)	0.036

Test used: univariate logistic regression; OR: odds ratio; CI: confidence interval; HF: health facility; ANV: antenatal care; TBA: traditional birth attendance; SBA: skilled birth attendance

According to the multivariate analysis of immunization status based on the health card plus history recalls, age group of 25-29 years (p=0.000) and no knowledge of immunization (p=0.000) significantly increased the odds of children being fully immunized, while the Murle ethnic group (p=0.036) and delivery assistance by SBA (p=0.000) significantly decreased the odds of children being fully immunized. On the other hand, age groups, including 25-29 years (p=0.000), 30-34 years (p=0.000), and 35-39 years (p=0.000) had positive impact on partial immunizations, while formal (p=0.000), delivery at a health facility (p=0.000), and delivery assistance by (p=0.000) had a negative impact on partial immunizations (Table 4, Table 4 (suite)). The most common reason given by mothers/caretakers in this study cohort for no immunization and partial immunization was the far distance of health facility (24.9%) followed by lack of knowledge (23.1%), fear of child abduction (15.8%), the mother being busy with family activities (15.3%), fear of side reaction from vaccination (11.4%), lack of service provider (5.5%), and bad behavior of the healthcare personnel administering vaccination (3.9%). Except for ethnicity and religion, there was a significant difference in the sociodemographic factors and the corresponding reasons for partial and no immunization status of the child (p<0.05). Lack of information was the common reason cited by participants age 20-24 years, ethnic group Jie, Kachipo, and Nuer residing in IDP/PoC, participants with education, housewives, and those who live 30 minutes from the health facility. While older participants (>30 years), employed participants, and those with no formal education reported distance as the main reason for non-immunization (Table 5).

**Table 4:** factors affecting the immunization status in Jonglei, South Sudan

	Card only										Card plus history recalls				
Variables	Fully immunized			Partially immunized			Not immunized			Fully immunized			Partially immunized		Not immunized
	OR (95%CI)	P value	OR (95%CI)		P value	OR (95%CI)		P value	OR (95	P value	OR (95%CI)	P value		OR (95%CI)	P value
									%CI)						
**Age**															
20-24	1(ref)		1(ref)			1(ref)			1(ref)		1(ref)			1(ref)	
25-29	0.021(0.008, 0.052)	**0**	0.147(0.076-0.284)		**0**	0.126(0.002-0.456		0.058	4.467(1.112-1.795)	**0**	9.011(2.349-3.456)	**0**		1.208(0.791-2.381)	0.291
30-34	1.466(0.000, 1.474)	0.997	0.021(0.006-0.075)		**0**	0.025(0.000-0.345)		0.78	4.774(0.000-5.128)	0.989	5.648(2.344-6.361)	**0**		0.392(0.000-1.248)	0.059
35-39	1.404(0.000-1.501)	0.998	4.518(0.000-5.012)		0.997	0.120(0.004-1.390)		0.89	1.369(0.000-2.391)	0.992	1.474(2.908-7.289)	**0**		2.381(1.282-3.971)	0.397
>40	1.404(1.404-1.404)	1	4.518(4.518-4.518)		1	0.529(0.257-3.908)		0.659	1.277(0.000-2.184)	0.996	1.207(0.000-1.843)	0.992		0.002(0.000-0.941)	0.057
**Ethnicity**															
Anyuak	1(ref)		1(ref)			1(ref)			1(ref)		1(ref)			1(ref)	
Dinka	0.711(0.228-2.220)	0.557	1.630(0.610-4.350)		0.33	1.290(0.258-2.589)		0.49	1.088(0.334-3.541)	0.889	1.200(0.423-3.401)	0.732		0.971(0.005-1.284)	0.284
Jie	8.483(8.483-8.483)	1	1.324(1.324-1.324)		1	1.418(1.418-1.418)		1	7.916(7.916-7.916)	1	1.123(1.123-1.123)	1		0.003(0.194-1.739)	0.428
Kachipo	5.655(0.000-6.210)	0.998	2.365(0.000-3.638)		0.998	0.235(0.002-2.589)		0.069	7.916(7.916-7.916)	1	1.123(1.123-1.123)	1		0.003(0.194-1.739)	0.428
Murle	8.280(0.000-9.316)	0.997	0.800(0.215-2.972)		0.739	1.218(0.439-2.797)		0.427	0.083(0.008-0.849)	0.036	0.787(0.232-2.675)	0.702		0.231(0.038-1.284)	0.742
Nuer	2.089(0.751-5.808)	0.158	1.259(0.475-3.335)		0.643	0.212(0.020-0.695)		0.068	1.481(0.471-4.579)	0.495	1.133(0.414-3.100)	0.807		0.481(0.024-1.285)	0.593
**Residence**															
IDP/PoC	1(ref)		1(ref)			1(ref)			1(ref)		1(ref)			1(ref)	
Own village	1.426(1.426-1.426)	1	1		1	0.021(0.000-0.649)		0.089	2.329(2.329-2.329)	1	1	1		0.320(0.023-0.581)	0.058
**Education**															
Primary	1(ref)		1(ref)			1(ref)			1(ref)		1(ref)			1(ref)	
Secondary	0.833(0.833-0.833)	1	3.315(0.000-5.862)		0.997	2.375(0.785-3.267)		0.692	1	1	1	1		1	1
College	1.000(1.000-1.000)	1	1.000(1.000-1.000)		1	0.217(0.016-1.025)		0.059	1	1	1	1		1	1
No education	2.716(0.000-3.015)	0.992	0.980(0.000-1.296)		1	0.025(0.000-1.349)		0.075	3.037(0.000-3.037)	0.997	1	1		1	1
**Occupation**															
No employment	1(ref)		1(ref)			1(ref)			1(ref)		1(ref)			1(ref)	
Formal employment	1.152(1.152-1.152)	1	1.152(1.152-1.152)		1	1.571(0.237-1.982)		0.089	2.163(2.163-2.163)	1	0.093(0.031-0.278)	**0**		0.391(0.029-1.834)	0.056
**Distance to HF**															
30minutes	1(ref)		1(ref)			1(ref)			1(ref)		1(ref)			1(ref)	
60minutes	0.030(0.004-0.244)	0.001	1.269(0.131-12.292)		0.837	1.912(0.021-4.912)		0.892	5.829(0.000-5.829)	0.99	6.284(6.284-6.284)	1		0.021(0.004-0.589)	0.385
>60minutes	8.772(8.772- 8.772)	1	0.010(0.001-0.099)		**0**	3.991(0.000-4.921)		0.782	9.049(0.000-9.049)	0.99	7.064(0.000-7.064)	0.99		0.073(0.004-1.578)	0.058

Test used: multivariate logistic regression OR- odds ratio, CI-confidence interval, HF-health facility, ANC-antenatal care, TBA- traditional birth attendance, SBAs-killed birth attendance

**Table 4(suite):** factors affecting the immunization status in Jonglei, South Sudan

	Card only						Card plus history recalls					
Variables	Fully immunized		Partially immunized		Not immunized		Fully immunized		Partially immunized		Not immunized	
	OR (95%CI)	P value	OR (95%CI)	P value	OR (95%CI)	P value	OR (95%CI)	P value	OR (95%CI)	P value	OR (95%CI)	P value
**ANC attendance**												
Yes	1(ref)		1(ref)		1(ref)		1(ref)		1(ref)		1(ref)	
No	5.565(5.565-5.565)	1	0.010(0.005-0.024)	**0**	0.089(0.000-6.491)	0.672	1.813(0.000-1.813)	0.979	1.574(1.574-1.574)	1	1.479(0.478-4.178)	0.628
**Delivery place**												
Home	1(ref)		1(ref)		1(ref)		1(ref)		1(ref)		1(ref)	
Health facility	5.126(5.126-5.126)	1	0.074(0.034-0.160)	**0**	0.014(0.002-3.285	0.247	4.133(4.133-4.133)	1	0.040(0.012-0.133)	**0**	1.285(0.029-2.583)	0.185
**Assisted by**												
TBA	1(ref)		1(ref)		1(ref)		1(ref)		1(ref)		1(ref)	
SBA	5.695(5.695-5.695)	1	0.107(0.052-0.218)	**0**	0.146(0.0120-6.185)	0.429	0.001(0.000-0.006)	**0**	0.035(0.011-0.116)	**0**	0.385(0.008-4.828)	0.385
**Postnatal services**												
Yes	1(ref)		1(ref)		1(ref)		1(ref)		1(ref)		1(ref)	
No	2.316(2.316-2.316)	1	0.015(0.006-0.036)	0.015	2.672(1.389-3.284)	0.385	1.944(0.000-1.944)	0.988	1.128(1.128-1.128)	1	2.581(0.027-3.578)	0.582
**Knowledge**												
Yes	1(ref)		1(ref)		1(ref)		1(ref)		1(ref)		1(ref)	
No	0.002(0.000-0.007)	**0**	0.020(0.010-0.044)	**0**	0.001(0.000-0.859)	0.067	1.578(1.438-4.579)	**0**	2.268(2.268-2.268)	1	4.829(0.481-5.721)	0.693
**Age of child**												
12-17 months	1(ref)		1(ref)		1(ref)		1(ref)		1(ref)		1(ref)	
18-23 months	4.538(4.538-4.538)	1	0.015(0.004-0.062)	**0**	2.479(1.573-3.581)	0.581	1.813(0.000-1.813)	0.979	3.111(3.111-3.111)	1	1.380(0.175-3.027)	0.286
**Gender of child**												
Male	1(ref)		1(ref)		1(ref)		1(ref)		1(ref)		1(ref)	
Female	2.693(2.693-2.693)	1	0.022(0.008-0.056)	**0**	0.948(0.481-1.489)	0.072	2.082(0.000-2.082)	0.979	6.067(6.067-6.067)	1	0.275(0.028-1.385)	0.138

Test used: multivariate logistic regression OR- odds ratio, CI-confidence interval, HF-Health facility, ANC-antenatal care, TBA- traditional birth attendance, SBAs-killed birth attendance

**Table 5:** correlation between maternal factors and reasons for partial and no immunization status of the child

Variable	Lack of information	Fear of side reactions	Vaccinator's behavior	Fear of child abduction	Family commitment	Distance	Lack of service provider	P value
Frequency	89 (23.1)	44 (11.4)	15 (3.8)	61 (15.9)	59 (15.3)	96 (24.9)	21 (5.5)	
**Mother's age**								0
20-24	84 (68.3)	24 (19.5)	4 (3.3)	11 (8.9)	0 (0.0)	0 (0.0)	0 (0.0)	
25-29	5 (3.3)	20 (13.2)	11 (7.3%)	49 (32.5)	44 (29.1)	21 (13.9)	1 (0.7)	
30-34	0 (0.0)	0 (0.0)	0 (0.0)	1 (1.4%)	15 (21.1)	50 (70.4)	5 (7.0)	
35-39	0 (0.0)	0 (0.0)	0 (0.0)	0 (0.0)	0 (0.0)	20 (66.7)	10 (33.3)	
> 40	0 (0.0)	0 (0.0)	0 (0.0)	0 (0.0)	0 (0.0)	5 (50.0)	5 (50.0)	
**Ethnicity**								0.047
Anyuak	5 (14.3)	4 (11.4)	1 (2.9)	8 (22.9)	6 (17.1)	9 (25.7)	2 (5.7)	
Dinka	20 (15.9)	16 (12.7)	5 (4.0)	24 (19.0)	21(16.7)	33 (26.2)	7 (5.6)	
Jie	2 (100.0)	0 (0.0)	0 (0.0)	0 (0.0)	0 (0.0)	0 (0.0)	0 (0.0)	
Kachipo	3(100.0)	0(0.0)	0 (0.0)	0 (0.0)	0 (0.0)	0 (0.0)	0 (0.0)	
Murle	6 (20.0)	4 (13.3)	1 (3.3)	0 (0.0)	5 (16.7)	8 (26.7)	6 (20.0)	
Nuer	53 (28.0)	20 (0.6)	8 (4.2)	29 (15.3)	27 (14.3)	46 (24.3)	6 (3.2)	
**Religion**								0.751
Christianity	88 (23.0)	44 (11.5)	15 (3.9)	61 (15.9)	58 (15.1)	96 (25.1)	21 (5.5)	
Muslim	1 (50.0)	0 (0.0)	0 (0.0)	0 (0.0)	1 (50.0%)	0(0.0)	0 (0.0)	
**Residence**								0
IDP/PoC	8 (100.0)	0 (0.0)	0 (0.0)	0 (0.0)	0 (0.0)	0 (0.0)	0 (0.0)	
Own village	81 (21.5)	44 (11.7)	15 (4.0)	61 (16.2)	59 (15.6)	96 (25.5)	21 (5.6)	
**Education**								0
Primary	19 (100.0)	0 (0.0)	0 (0.0)	0 (0.0)	0 (0.0)	0 (0.0)	0 (0.0)	
Secondary	6 (100.0)	0 (0.0)	0(0.0)	0 (0.0)	0 (0.0)	0 (0.0)	0(0.0)	
College	1 (100.0)	0 (0.0)	0 (0.0)	0 (0.0)	0 (0.0)	0 (0.0)	0 (0.0)	
No education	63 (17.5)	44 (12.3)	15 (4.2)	61 (17.0)	59(16.4)	96(26.7)	21 (5.8)	
**Employment**								0
No	89 (24.2)	44 (12.0)	16 (4.1)	61 (16.6)	59 (16.0)	83 (22.6)	17 (4.6)	
Yes	0 (0.0)	0 (0.0)	0 (0.0)	0 (0.0)	0(0.0)	13(76.5)	4 (23.5)	
**Travel time**								0
30 minutes	52 (96.3)	1 (1.9)	0 (0.0)	0 (0.0)	0 (0.0)	0 (0.0)	1 (1.9)	
60 minutes	35 (35.7)	36 (36.7)	13(13.3)	14(14.3)	0(0.0)	0 (0.0)	0 (0.0)	
> 60 minutes	2 (0.9)	7 (3.0)	2 (0.9)	47(20.2)	59(25.3)	96 (41.2)	20 (8.6)	

Test used: chi square test; values are presented in n (%)

## Discussion

In this study, only 17.7% of children between the ages of 12-23 months were fully immunized, when the data was verified using health cards. It increased to 27.5% when the data was verified using both cards and mothers' history recalls. The difference could be due to the inability of the mother to recall the exact schedules; since the immunization program consists of many sessions, the mother/caretaker may overestimate the vaccines received by the child. Keeping records of vaccination by electronic media may eliminate the risk of recall bias and give accurate data. Although the vaccination coverage is much higher than the 7.3% reported by Mbabazi *et al*. [7] in South Sudan, the immunization coverage is much lower than those reported in other countries, including 33% in Nigeria [9], 53% in Myanmar [10], 69.21% in East Africa [11], 59% in Lao People´s Democratic Republic [12], 45.7% in Afghanistan [13], and in 84.5% in Ghana [14]. While Daniels *et al*. [15] have reported higher immunization rates among male children, other researchers did not observe a significant difference between gender and immunization [12,13,16-18]. The rate of full immunization was higher in the younger age group and higher in males and in those children who were ill 2 weeks before the vaccination schedule. Interestingly, none of the girl children were fully immunized. The rate of individual immunization in this study (58-77%) is much lower than those reported by Mbengue *et al*. [19] (>80%) and higher than reports by Tamirat and Sisay [20] (50-60%). Like reports by Adedire *et al*. [21], the rate of initial vaccine doses is higher (70-77%) as compared to the vaccines scheduled later (50-60%). It could be because the BCG and the initial vaccines are administered right after birth, while the later sessions are dependent on the parent´s cooperation which reflects the poor compliance and forgetful appointments by parents [17].

Evidence suggests that mother´s socioeconomic status, maternal age, ethnicity, education, employment, ANC and PNC visits, place of delivery, birth assistance, wealth index of the family, TT immunization, access to services, short travel time, knowledge of immunization, child´s age, number of children in the family, preceding birth interval are significant predictors of immunization [12,12,19-25]. In this study, the frequency of vaccination was higher in mothers less than 30 years, specifically those between 25-30 years, as compared to mothers above 30 years. This could be related to the acquired knowledge about the benefits of the vaccination by the mothers [11,26]. Similar to Xeuatvongsa *et al*. [12], ethnicity also played an important role in immunization status in Jonglei. Similar to reports of Ismail *et al*. [16], immunization coverage was higher in children living at IDPs/PoC as compared to children living in the village (100.0% vs. 15.9%), which is correlated to better access to health services in the protection camps. Maternal education and level of literacy have a positive impact on the child's immunization status [12,27]. Forshaw *et al*. [28] in their systematic review of 37 studies, conferred that child of an educated mother has 2.3 times higher odds of getting all scheduled vaccinations as compared to a child whose mother had no education. Similarly, immunization coverage was higher in children of educated mothers than mothers with no formal education (>80% vs. 12.05%). Similar to Tefera *et al*. [18], immunization was higher in children of unemployed mothers. I believe since the study was in a rural setting, employment merely meant agriculture, and those mothers, due to the busy work life, would have missed the vaccination appointments. Travel time from home to health facilities is inversely related to the vaccination coverage agrees with these results [12,18,29].

In accordance with previous research, a positive correlation between the utilization of health facilities by mother, including the number of ANC visits, delivery at the health facility, and PNC visits with immunization status [21,22,29-31]. This could be correlated to the counseling and education session on immunization given during the visit to the health facility. On the other hand, immunization coverage was higher in children born at home than those born at a health facility. It may be because of the cultural beliefs of the communities who feel safer at home during childbirth [32]. In contrary to the studies suggesting a positive correlation between SBA and immunization [33,34], immunization coverage was higher in children assisted by TBA in this study. As suggested previously [21,35], tetanus immunization taken during pregnancy was significantly associated with child immunization coverage. Maternal knowledge about immunization programs and the associated vaccines increases the likelihood of the routine immunization of their child [21,36,37]. Although only 35.1% had heard about immunization in this study cohort; nevertheless, knowledge had a positive correlation with a child´s immunization status. In contrary to the male partners being considered barriers of child immunization [38,39], information obtained from husbands led to an increased rate (94.4%) of immunization.

Previous studies have suggested that lack of knowledge is one of the main reasons for low immunization [7,16,39,40]. Other reasons include vaccine-related reactions [41], vaccine provider´s hostility and rude attitude [42], vaccine hesitancy [43], fear of side effects, busy schedules, poverty, long distance between home and health facility, non-availability of vaccine, long waiting time, forgetfulness, inconvenient time, and language barrier [13,16,18]. In this study, the most common reason for no immunization and partial immunization was a far distance of health facilities (24.9%) followed by lack of knowledge (23.1%). To overcome the knowledge related barrier, it is recommended to increase health education by means of community meetings, radio and mass media. It is advisable to increase the number of health facilities, especially in remote areas, with reliable staff, and to educate them to be kind to avoid hostility towards the visiting patients. Increasing the days of vaccination in a health facility and creating more outreach programs, or integrating immunization programs with other health-related services like nutrition may have a positive impact. Community-based study and the data collection based on personal interviews of the mothers/caretakers by trained healthcare workers/volunteers who endured accurate data recording is the main strength of the study. However, the cross-sectional nature of the study does not allow evaluating the cause-and-effect relationship between immunization and risk factors. Also, since most of the time, a health card was not available, the interviewer had to rely on the mother´s recall history, which could have been biased due to various reasons related to recall bias, overestimation, and community-related. Similarly, the validation of individual vaccines is difficult. Immunization service-related factors such as vaccine availability, presence of healthcare personnel, access to the health care facility, and its related factors were not assessed. Furthermore, factors like parity, birth spacing between children, and size of the family were not included in the present study, which could be included in future studies. Further research is warranted by including divisions of religious congregations as each will have practices and beliefs of its own.

## Conclusion

In this study, maternal age, child's gender, education, travel time, ANC, and PNC visits were significant predictors of immunization. Effective health education, access to healthcare services, and close monitoring of pregnant women and children (till 1 year of age) may improve the immunization status in Jonglei state, South Sudan. Having access to health information may play a pivotal role in increasing mother's knowledge on immunization and other health schemes. Furthermore, setting up more health facilities with qualified staff will further enhance immunization coverage. Needless to say, better immunization coverage is the result of the combined effort of policy makers, the healthcare system, local community leaders, and community health workers at the grass-root level and also the responsibility of individual parents.

### What is known about this topic

- Consistent low routine immunization coverage;
- Lack of previous research data.

### What this study adds

- The data will be used to better understand the routine immunization coverage;
- It will help the State Ministry of Health to design interventions;
- Inform decision for improvement of immunization coverage in the state.
